# Explicit metrics for implicit emotions: investigating physiological and gaze indices of learner emotions

**DOI:** 10.3389/fpsyg.2024.1440425

**Published:** 2024-12-13

**Authors:** Sharanya Lal, Tessa H. S. Eysink, Hannie A. Gijlers, Bernard P. Veldkamp, Johannes Steinrücke, Willem B. Verwey

**Affiliations:** Departent of Learning, Data-Analytics and Technology, Faculty of Behavioural, Management and Social Sciences, University of Twente, Enschede, Netherlands

**Keywords:** emotion recognition, learner emotions, physiological signals, eye-tracking, sensors, wearables

## Abstract

Learning experiences are intertwined with emotions, which in turn have a significant effect on learning outcomes. Therefore, digital learning environments can benefit from taking the emotional state of the learner into account. To do so, the first step is real-time emotion detection which is made possible by sensors that can continuously collect physiological and eye-tracking data. In this paper, we aimed to find features derived from skin conductance, skin temperature, and eye movements that could be used as indicators of learner emotions. Forty-four university students completed different math related tasks during which sensor data and self-reported data on the learner’s emotional state were collected. Results indicate that skin conductance response peak count, tonic skin conductance, fixation count, duration and dispersion, saccade count, duration and amplitude, and blink count and duration may be used to distinguish between different emotions. These features may be used to make learning environments more emotionally aware.

## 1 Introduction

Learning experiences are greatly influenced by the emotions of a learner (Pekrun, 2006). Therefore, it is pertinent that educators, designers, and researchers consider a learner’s emotions while creating learning systems that offer personalized support. For this to be possible, the first step is to be able to perceive a learner’s emotions and ideally in a continuous and non-obtrusive manner. This is what we address in the present paper.

### 1.1 Emotions in learning environments

There is a large body of work on the interaction of learner emotion and learning. For example, Csikszentmihalyi’s (1990) seminal work on ‘flow’ suggests that optimal learning experiences occur when individuals are in such a state of concentration that they lose track of time. This is often accompanied by deep enjoyment and happiness. An example is Pekrun et al.'s (2017) longitudinal study on the development of mathematical competencies of adolescents (grades 5 to 10) that found that enjoyment and pride positively predicted subsequent annual assessment scores; the converse was found to be true for anger, anxiety, shame, hopelessness and boredom. Boredom in particular has been found to be persistent in learning situations of durations ranging from 30 to 75 min and is associated with ‘gaming the system’ (in which students simply manipulate the system to succeed at a task instead of actually learning the content) (Baker et al., 2010) and poor learning (Baker et al., 2010; Craig et al., 2004). According to D’Mello and Graesser (2012), students in a state of flow experience confusion when faced with an obstacle to their goals, but their state of flow is restored if they are able to solve their problem. If they cannot, this confusion makes way for frustration and then boredom. So, while one cannot assert that optimal learning experiences consist solely of positive emotions (such as deep enjoyment and happiness), current research suggests that persistent negative emotions do affect learning negatively. Therefore, it is expected that learning systems that detect these emotions in order to adapt their support will provide optimal learning experiences.

### 1.2 Emotion detection

Research into emotions has traditionally used self-reported data (Wu et al., 2016). Apart from the obvious subjective nature of self-reports, this approach is also limited by the temporal mismatch between when an emotion is experienced and its corresponding data are collected (Yadegaridehkordi et al., 2019). Moreover, if one’s aim is to develop a system that can detect and adapt to emotions, it is impractical to constantly interrupt the learning process to ask the learner for input. This calls for objective, time-specific and unobtrusive data collection. Wearable and portable sensor technology today makes this possible because emotions are accompanied by physiological and behavioral responses. For example, people can find themselves with sweaty palms or a racing heart when extremely anxious. Sometimes people find themselves “wide-eyed” with surprise or shaking with fear or anger. Physiological and movement sensors can detect these signals, and using the appropriate techniques, one may make inferences about the associated emotion.

### 1.3 Sensor data in educational and emotion research

In their review of physiology-based mobile educational systems, Hernández-Cuevas and Crawford (2021) found that one of the most used measures, after eye-tracking, was heart rate (i.e., the number of heart beats per minute). In line with this, Liu et al. (2022) systematically reviewed learning analytics based on wearable devices, examining 120 articles published between 2011 and 2021, and found that heart rate and skin conductance (i.e., the level of perspiration in response to an emotional stimulus) were two of the most widely used sensor data. Heart rate has been included in several studies such as ones that investigated measures of mental workload (Sharma et al., 2020), student interaction (Darnell and Krieg, 2019) and cognitive load (Larmuseau et al., 2020). Among other things, skin conductance has been studied to profile sympathetic arousal of students during a physics class (Pijeira-Díaz et al., 2018), identify momentary student engagement in an afterschool program (Lee et al., 2019) and measure mental workload (Sharma et al., 2020). In emotion research specifically, skin conductance and heart rate have been investigated in the context of fatigue and drowsiness (Adão Martins et al., 2021). In the field of emotions during learning, some studies have found that skin conductance reflected stress (Brouwer et al., 2017), emotional arousal (i.e., the strength of an emotional state) (Jindrová et al., 2020), anxiety (Harley et al., 2019; Meer et al., 2016) and shame (Harley et al., 2019). In more recent preliminary explorations of emotions during parent–child learning activities, Avelar et al. (2022) and Shaby and Bokhove (2023) found that skin conductance could be used to discern different emotions. In their meta-study on test-anxiety and measures of physiological arousal, Roos et al. (2021) found that both skin conductance and heart rate significantly increased with self-reported test anxiety. However, there is no clear consensus yet on how exactly these signals vary with different emotions or how much variance they can explain. For example, Van Bruinessen et al. (2016) found no significant relationship between self-reported anxiety and skin conductance. In another study, Ritz et al. (2005) investigated physiological response to the viewing of pictures from IAPS (International Affective Picture System) and found that while heart rate significantly increased for both negative and positive emotions, there were no considerable changes in skin conductance. Nevertheless, a review by Ba and Hu (2023) showed that skin conductance and heart rate were two of the most studied measures of autonomic nervous system activity associated with emotions, and available evidence suggests that there is value in further exploration.

However, Kreibig (2010) warned that progress in research was hindered by the “exclusive use of convenience measures such as HR [heart rate] and electrodermal activity, as sole indicators of the activation state of the organism” (p. 409) and that it is essential to select more measures to determine patterns. Skin temperature is one such measure (Adão Martins et al., 2021; Noroozi et al., 2020). In their study with female undergraduate students, Rimm-Kaufman and Kagan (1996) found that hand skin temperature increased while watching film clips designed to induce happy affect and reduced when asked threatening personal questions. In a similar vein, McFarland (1985) found that music that was perceived as inducing negative emotions stopped an increase and perpetuated a decrease in skin temperature; calming music had the opposite effect. On the other hand, Jang et al. (2015) found that skin temperature increased with boredom (a negative emotion). Lal et al.’s (2021) study physiological correlates of learner emotions during different programming tasks suggested the same. Meanwhile, Jang et al. (2019) investigated the reliability of skin temperature as a response to different emotions and found it to be an unreliable indicator. Mixed results from past studies warrant further investigation into skin temperature as an indicator of emotions.

A relatively new approach in emotion research is tracking eye movements (Lim et al., 2020). Eye-tracking for emotion detection has usually been used in combination with physiological signals and there is mounting evidence that this is indeed useful (Lim et al., 2020). An example of this is Aracena et al.'s (2015) use of neural networks on multimodal data that included blinks and saccades (quick eye movements between fixations) to differentiate between negative, neutral and positive emotions. Other examples of successful use of eye-tracking include the use of gaze features for emotion recognition in patients with mesial temporal lobe epilepsy (Gomez-Ibañez et al., 2014) and in individuals in the autism spectrum (Tsang, 2018). While a wide variety of features has been used in the past, studies do not concur on which are the most effective for emotion recognition (Lim et al., 2020), and hence this warrants further research.

### 1.4 Present study

As outlined earlier, despite extensive research on skin conductance and heart rate as indicators of emotions, past results are varied. On the other hand, skin temperature despite being an easily accessible physiological measure that could be used as an emotional indicator, has rarely been investigated with respect to learner emotions (Noroozi et al., 2020). Moreover, eye-tracking has only recently been included in emotion detection and here too, findings are inconclusive. More interestingly, Noroozi et al. (2020) found that out of the 207 publications included in their systematic review of multimodal metrics to capture the learning process, only 15 included the emotional aspect of learning. This imbalance in past literature underscores the need for further research that focuses on (under represented) indicators of emotions, specifically of learners. In the present study, we investigated skin conductance, heart rate, skin temperature, and eye movement metrics as indices of learners’ emotions. To this end, we adopted a dimensional approach to emotions based on Russell’s (1980) widely accepted circumplex model of emotions, which posits that emotions may be represented along two orthogonal dimensions, arousal and valence. Emotional arousal may be defined as the activation level or strength of an emotion, while emotional valence is its hedonic nature (Pekrun, 2006; Russell et al., 1989; Thayer, 1967; Thayer, 1978). Consequently, emotions fall into any one of the four quadrants determined by the axes arousal and valence – high arousal-negative valence (for example, frustration), high arousal-positive valence (for example, happiness), low arousal-positive valence (for example, calmness) and low arousal-negative valence (for example, boredom). For a visual representation of different emotions on a two-dimensional scale, see Figure 6 of Russell (1980). Our research was motivated by the need to develop emotionally aware systems that can encourage positive and reduce persistent negative emotions during learning. Therefore, in the present study we investigated relevant (and unobtrusive) indices of the four emotional quadrants. Learner emotions were indexed by self-reported arousal and valence. In line with the literature cited earlier, we investigated the prospects of using the following three measures of physiological arousal – skin conductance, heart rate and skin temperature, and features derived from all three events that take place during eye-movement (Hessels et al., 2018) – blinks, saccades and fixations. It is important to note that the selection of measures was based also on the possibility of using them in real-world classrooms. The study was quasi-experimental and involved data that was collected at regular intervals during multiple math-related tasks in a counterbalanced set-up.

## 2 Methods

### 2.1 Participants

Participants consisted of 44 (32 females and 12 males, 18–26 years old, *M*_age_ = 20.09 years, *sd* = 1.89, 40 right-handed and 2 left-handed) bachelor’s students from the Faculty of Behavioural, Management and Social sciences at the University of Twente (the Netherlands). The sample consisted of persons of 12 nationalities, namely: Bulgarian (*n* = 1), Brazilian (*n* = 1), Chinese (*n* = 1), Croatian (*n* = 1), French (*n* = 1), German (*n* = 21), Greek-German (*n* = 1), Malaysian (*n* = 1), Dutch (*n* = 11), Dutch-German (*n* = 1), Dutch-Ukrainian (*n* = 1) and Romanian (*n* = 3). All participants had at least working knowledge of English. Participants were recruited through an online participant management system. Participation was voluntary in exchange for 2 study credits. All participants had provided informed consent to participate in the study, which included the collection of demographic, physiological, eye-tracking and self-reported data.

### 2.2 Materials

#### 2.2.1 Tasks and baseline stimulus

**Figure 1:**
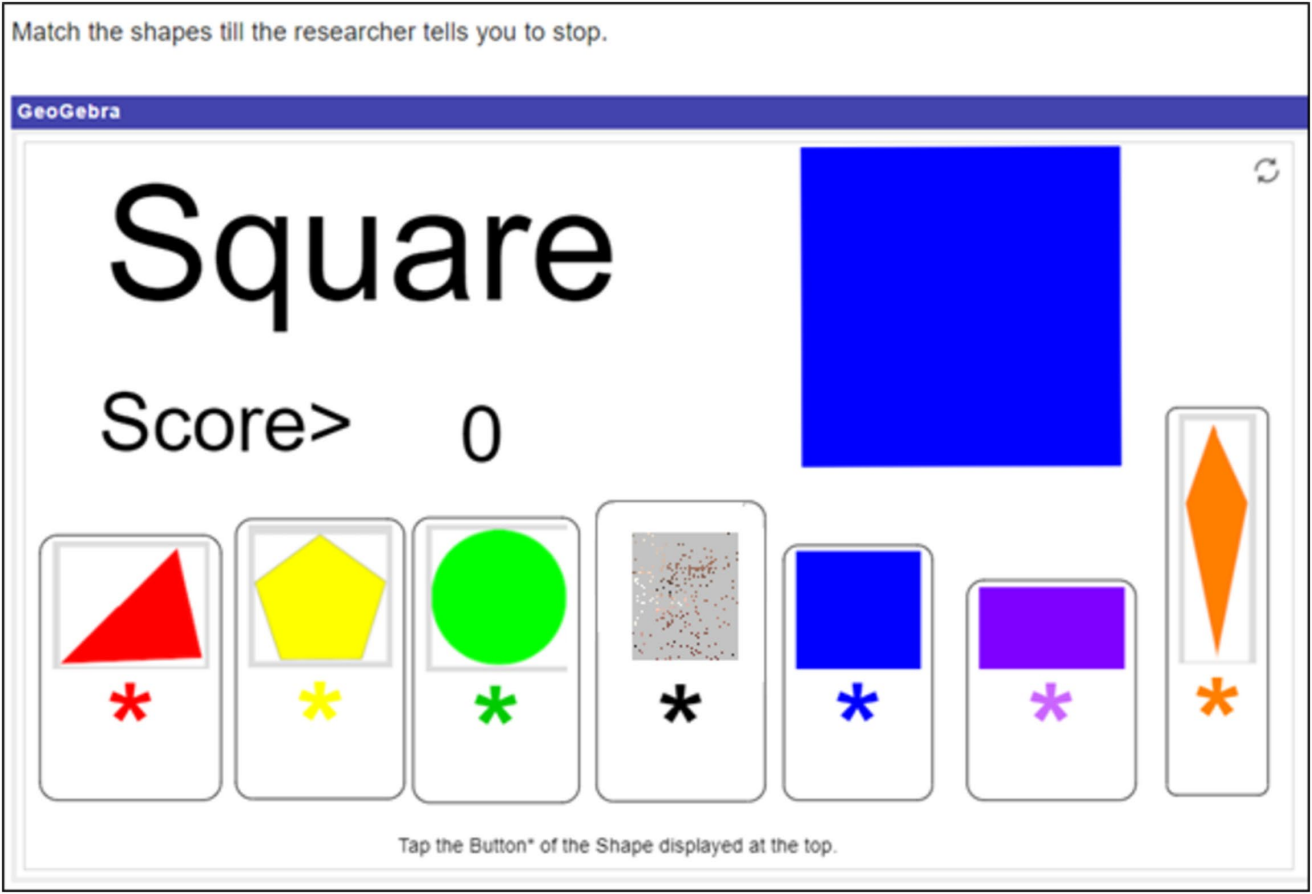
The shape matching task. In this instance, clicking on the blue square in the row would increase the score by 1 point. Created with GeoGebra^®^, by Lindenmuth https://www.geogebra.org/m/rvz58cma#material/eMCXcErd.

**Figure 2:**
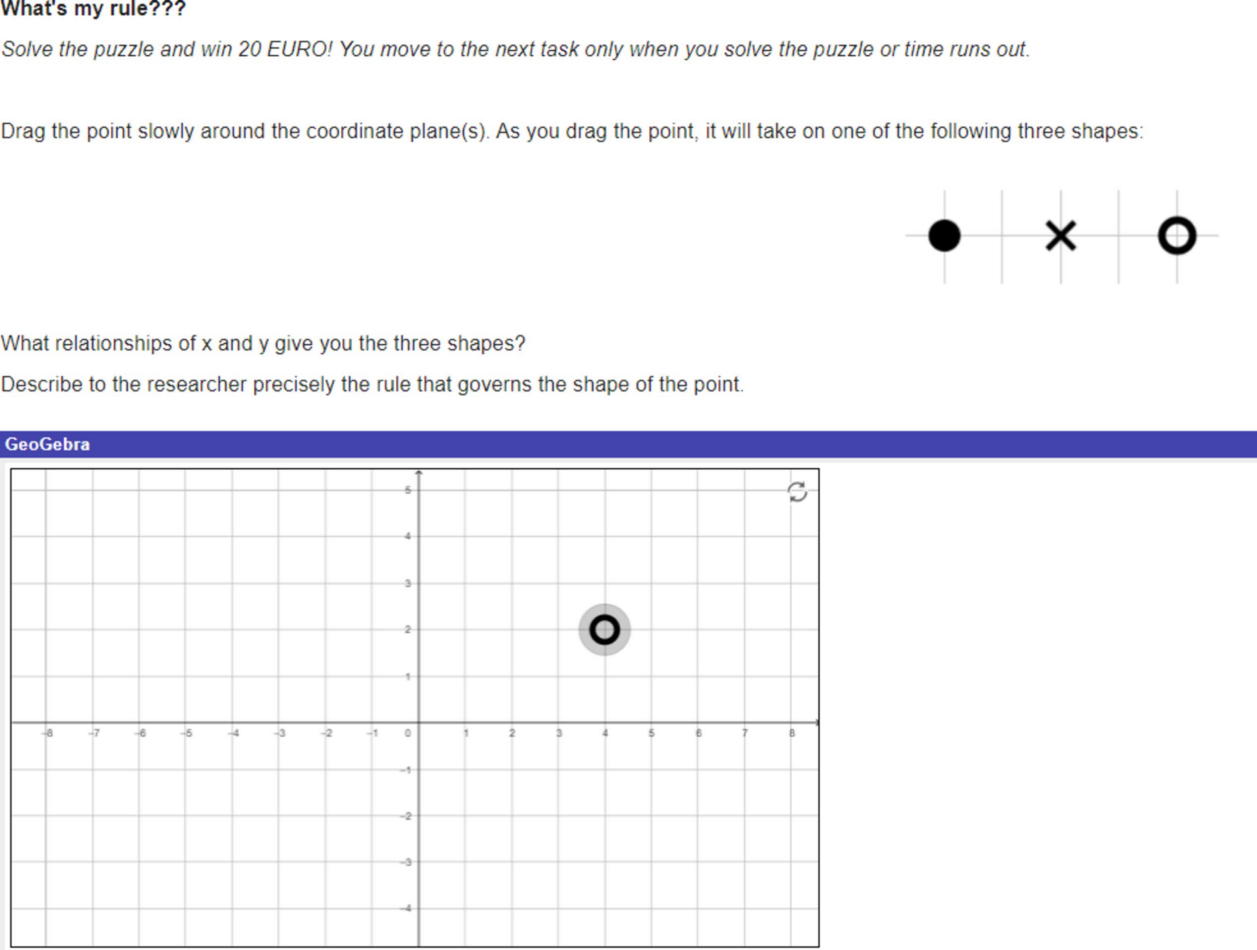
The coordinate geometry puzzle. Created with GeoGebra^®^.

Participants engaged with three different math related tasks. All tasks were designed such that they could be done with just one hand. This was to mitigate motion artefacts in signal data from the wearables used in the study. The first task henceforth called the *shape matching task*, utilized an elementary school level math simulation called “Shapes Matching: Scored” (Lindenmuth, n.d.; Figure 1). In each round, a two-dimensional shape was displayed at the top of the screen. Participants were required to scan the row of shapes below the presented one and click on the matching shape. Instructions included no indication of how long the task would last and all points scored were inconsequential. While there were indefinite rounds in the task, participants completed only as many as was possible in 12 min. The second task was a high school level *coordinate geometry puzzle* (Figure 2). Participants were required to move the cursor to move the point (marked by the orange arrow) along the coordinate plane in order to find “the rule that governs the shape of the point.” The rule was that the point changed shape based on whether it lays inside, on, or outside a parabola. Instructions stated that the activity would only end when participants solved the puzzle (to win €20), or time ran out (with no indication of when exactly that would be). However in reality, this task too ended after 12 min. Participants thus had an indefinite number of attempts at solving the puzzle. Irrespective of what answer the participant gave, the researcher told them they were wrong, essentially making this a frustrating activity. The third task was online (Tetris, 1985). Tetris is a video game in which players attempt to complete rows of a grid by arranging differently shaped tetrominoes that fall onto the playing field. The game has been found to bring players into a positive emotion of effortless attention (Harmat et al., 2015). It is not only widely popular among gamers and recreational mathematics enthusiasts, but is also a widely studied game (Mayer, 2014), drawing interest from psychologists, mathematicians, and computer scientists (e.g., Chanel et al., 2008; Maier et al., 2019; Tsuruda, 2010). Tetris (and its variations) has been found to improve algorithmic thinking and spatial skills and is often used to teach geometrical concepts such as rotation, translation and reflection (Clements et al., 1997; Sims and Mayer, 2001; Williams and Bright, 1991). In an attempt to foster engagement in this task, participants were offered prize money based on their Tetris score. Participants won €5 for the first 20,000 points in Tetris. For every 10,000 points after that, they won €1. If they made it to the leader board, they won additional money – up to €5 – depending on their rank. (Twenty-three participants won on average €6.52 (*sd* = 3.46), with the highest prize money being €22). Participants knew they had 12 min to play and were free to restart the game any number of times within that period.

In an effort to bring participants to more or less the same starting point in terms of their emotional state, participants were instructed to “Sit still. Relax and clear your mind while watching this relaxing video” of an underwater scene with calm instrumental music (4 min). The decision to not use a math-related baseline was a conscious one – the baseline was intended to elicit minimal stimulation without priming participants to the math-related nature of the study. The use of this video is a deviation from the traditional ‘resting baseline’ in which participants usually do not engage in any activity and stay in a relaxed or ‘resting’ state. Research finds that engaging in an activity that requires minimal cognitive effort minimizes intrusive thoughts and produces results equal to or better than resting baseline conditions (Jennings et al., 1992). In fact, Piferi et al. (2000) found that watching a relaxing aquatic video produced lower (cardiovascular) baseline levels than traditional methods.

The tasks, baseline stimulus and self-reports (described below) were finalized after three rounds of iterative pilot testing involving a total of 13 pilot participants. Based on researcher observations and participant responses, we expected participants to report low arousal positive emotions while watching the baseline video, low arousal negative emotions over time while matching shapes, high arousal negative emotions over time doing the coordinate geometry puzzle, and high arousal positive emotions playing Tetris.

#### 2.2.2 Measures and instrumentation

Self-reported measures were collected using the Affect Grid (Russell et al., 1989), a 1-item scale of emotions along the two dimensions, emotional arousal (Cronbach’s *α* = 0.81) and valence (Cronbach’s α = 0.79), both of which could range from values 1 to 9. The affect grid was selected because it is a quick capture tool and is appropriate for repeated measurements (Killgore, 1998). Respondents check a square on the 9×9 grid to indicate their emotion along two dimensions, valence (along the X-axis) and arousal (along the Y-axis). The mid-point of the grid is (5,5) which denotes neutral valence or arousal. High arousal-negative valence, high arousal-positive valence, low arousal-positive valence and low arousal-negative valence emotions are marked in the first, second, third and fourth quadrants, respectively. An open-ended fill-in-the-blank statement “I am feeling __” was used to verify that the participant had in fact thought through the filling of the affect grid. This self-report was administered every 4 min.

Four physiological measures were assessed. Skin conductance and heart rate were measured using the Shimmer3 GSR+, a biosensing unit with an average sampling frequency of 128 Hz. Skin conductance was measured between two stainless steel electrodes attached to the palmar region of two fingers. Heart rate was derived from a photoplethysmogram signal collected by a pulse probe clipped to the ear. Peripheral skin temperature was measured with the infrared thermopile sensor (sampling rate of 4 Hz) of the Empatica E4 biosensing wristband. Eye-tracking was done using Tobii Fusion Pro, a screen-based eye-tracker with sampling frequencies up to 250 Hz. Its camera was attached horizontally at the bottom of the computer screen the participants’ tasks were displayed on.

Several supporting tools were used in the study. The Tobii Fusion Pro and the Shimmer3 GSR+ were configured using Tobii’s eye-tracking manager and Shimmer’s ‘Consensys’ software. These were synchronized on iMotions, an integrative software platform for biometric research. iMotions was used to time and present the tasks and instructions, and collect, visualize, and pre-process data from the two devices mentioned above. Calibration of the eye-tracker for each participant was also done on iMotions using a 9-point calibration slide; light calibration was done using a single grey screen. Skin temperature data were streamed to Empatica’s cloud-based repository via an android application set up on a mobile phone which in turn was connected via Bluetooth to the Empatica E4. Other pieces of equipment used were a 24 inch AOC G2460PF computer monitor (refresh rate 144 Hz) used as the participant’s primary screen, a Microsoft Surface Pro touchscreen tablet used to record participants’ self-reports, a wired mouse for the use of the participants and two HP Elitebook laptops (64-bit operating system, Intel(R) Core(TM) i5-8250U processor with CPU @ 1.60GHz) – one that served as the researcher’s primary device (used to initiate and monitor the study on iMotions) and the other for the researcher to note down observations. A Jellycomb 1920 × 1,080-pixel webcam was mounted on the top-centre of the participant’s computer to collect face recordings. These were to be used to explain missing eye-tracking data if any. A room thermometer was used to record ambient temperature at the start of the experiment.

### 2.3 Procedure

Before the experiment, participants (wherever applicable) tied or pinned up long or loose strands of hair, removed makeup and accessories from their wrists and ears, and rolled up their sleeves. They were individually seated in a closed, well-lit and thermoregulated room (average ambient temperature 25 degrees Celsius). After completing an informed consent form and a demographics questionnaire, they received a general outline of the experimental set-up, procedure, tools, and expected code of conduct. The Affect Grid specifically was explained in detail – arousal was described as “how activated or aroused you feel” while pointing at the y-axis on the Affect Grid and valence was described as “how unpleasant or pleasant your emotion is” while pointing at the x-axis. This was followed by check-for-understanding (CFU) questions such as “You are running late for an exam and your bike has a flat tyre. Where would you mark an ‘X’ on the grid?” and a quick think-aloud of the reporting. For example, if a participant pointed to the first (high arousal-high valence) quadrant, the researcher would say something along the lines of “Yes, maybe because you feel anxious and anxiety is a negative emotion that is also activating” Other CFU questions/scenarios used were “You are thinking about a party you will attend this evening with your friends,” “You just did yoga/meditation and are feeling relaxed” and “You are sitting in a very boring lecture and falling asleep.” Next, participants were informed that all the instructions they would need would be on the screen and that the researcher would not help them with the tasks. They were also informed that if there was prize money attached to a task, this information would be in the task’s instructions – textual information about prize money preceded the coordinate geometry puzzle and Tetris.

The Shimmer3 GSR+ unit was strapped tightly to the non-dominant forearm to mitigate motion artefacts. Its electrodes were attached to the third and fourth proximal phalanges of the participant’s non-dominant hand. The ear clip was attached to the corresponding earlobe. Participants were also fitted with an Empatica E4 on the same hand making sure that the thermopile sensor made complete contact with the dorsal side of the hand. Once the wearables were switched on and streaming data, participants waved their hand around a few times while the researcher ran a visual check on the signals. Thereafter, participants sat still for at least 10 min while signal readings were checked. This was done to ensure that the electrodes coupled with the participant’s skin before the start of the experiment.

Participants were seated at a distance between 60 cm and 70 cm from the computer screen such that (a) they could see the reflection of their nose on the eye-tracker’s frontal surface (an indication that the eye-tracker was at an optimum distance and height) and (b) iMotions’ ‘eye finder’ widget indicated that both eyes were being detected. Participants placed their non-dominant hand on their lap and were discouraged from fidgeting or making big motions during the study.

The experiment was set up on iMotions meaning that all sensors were synchronized, and stimuli were timed and displayed on the platform. The study commenced when the quality of the eye-tracker calibration was deemed “excellent” by iMotions. The experiment started with a baseline reading (4 min) after which participants performed the three tasks. Each task lasted 12 min and participants made multiple attempts at the tasks during these periods. The order of the three tasks was counterbalanced across participants. Every four minutes, participants paused to complete the self-report on the touchscreen tablet. Thus, 13 instances of the self-report were collected – one for the baseline and three for each task. Participants had a 1-min ‘cooling off’ period between tasks while a screen with the instructions ‘Sit still, relax and clear your mind’ was displayed on their monitor. Participants were debriefed at the end of the experiment.

### 2.4 Signal processing

Most studies using skin conductance split the signal into the phasic component (i.e., the fast-moving signal that is an immediate response to stimuli) and the tonic component (i.e., the slow-moving signal) (Horvers et al., 2021). However, there is no consensus on which component to use (Horvers et al., 2021). Therefore, we investigated both. We used iMotions’ R notebooks with their default parameters to process the raw skin conductance signal (measured in μS) (iMotions, 2022a). A time window of 4,000 ms was set as the threshold to determine gaps in the signal (due to signal drops) that would be linear interpolated. Missing data in gaps longer than the threshold were not interpolated and the resulting signal fragments were processed separately.

The phasic component was separated from the tonic component using a median filter over a time window of 8,000 ms. A lowpass Butterworth filter of 5 Hz was applied for noise filtration of the phasic signal. Skin conductance response (SCR) peaks were extracted from the phasic component using a 0.01 μS peak onset threshold, a 0 μs offset threshold and a 0.005 μS peak amplitude threshold. An onset is when the phasic signal surpasses a predetermined onset threshold, and an offset is when the signal drops below an offset threshold. A peak is the maximum value of a phasic signal within a time window determined by an onset-offset pair. Its amplitude is calculated as the difference between the value at the highest point and the value of the phasic signal at the onset. Each onset-offset pair defines a window, and the maximum value attained by the signal in this window is considered a peak. A value was marked as a peak if it was above the amplitude threshold of 0.005 μs in a window longer than 500 ms.

Eye-tracking data were processed using iMotions’ R notebooks using their default parameters (iMotions, 2022b). Blinks were registered when data for both eyes were lost (an indication that eyes are closed) between 20 ms and 500 ms. Blinks were merged when the time between them was less than 70 ms. Fixation and saccade features were extracted using an I-VT (velocity-threshold identification) filter – if eyes moved slower than a velocity threshold of 30 degrees per second, the event was classified as a fixation and if they moved faster, a saccade was recorded. Fixation dispersion (i.e., the spread of a fixation’s gaze points) was calculated by Imotions as the root mean square of the samples belonging to that fixation. Gaps in the signal shorter than 75 ms were interpolated.

Heart rate was calculated within iMotions. Linear interpolation was used to fill these gaps in the signal if the percentage of invalid data points was less than 10%. Since skin temperature was not collected in iMotions, it was synchronized (post-experiment) with the tasks using a Python script. Visual checks indicated no missing data.

For all participants, skin conductance and eye-tracking data with less than 20 dB signal-to-noise ratio (as indicated by iMotions) and all signals with greater than 10% missing data were excluded from the analysis. This resulted in excluding HR data of 10 participants from the analysis.

All in all, the following 13 data features were extracted – SCR peak count, average SCR peak amplitude, tonic skin conductance level, fixation count, duration and dispersion, saccade count, duration and amplitude, blink count and duration, heart rate, and skin temperature.

### 2.5 Analysis

To address issues of subjectivity and individual physiological variability, standardized values of physiological and gaze measures were used – mean values for the four-minute windows corresponding to each self-report (from the baseline and the three tasks) were calculated and finally z-scores *per participant* were computed. Self-reported emotions were labeled 1–4 based on the Affect Grid quadrant they fell in. For example, an ‘X’ on position (7,9) of the Affect Grid was labeled as 2 because it was in the second quadrant. Signals labeled with similar emotion labels (i.e., in the same quadrant) could come from different tasks, meaning that a label did not represent a task exclusively. Data points on the axes and therefore not in any quadrant [for example (5,7) or (8,5)] were excluded from further quantitative analyses. Finally, to determine if signals varied across emotional quadrants and if yes, what the pairwise differences were, Kruskal-Wallis tests with *post hoc* Dunn tests were performed. Since the baseline video was intended solely to provide a common point of departure for all participants and was therefore not included in the study’s counterbalancing design, baseline readings were used for the computation of z-scores; however, they were excluded from the pairwise comparisons. This approach of using within-person z-scores provides a more reliable method of handling variability as compared to making calculations relative to a single baseline, because it acknowledges that: (a) a person’s ‘baseline’ can shift due to various factors, making it difficult to capture a “true” baseline in a single measurement, (b) a single baseline measurement may not fully reflect an individual’s actual physiological state and (c) subjective experiences of baseline tasks vary, and it is difficult to bring all participants to true ‘baseline levels’ with one standardized task.

## 3 Results

### 3.1 Self-reported emotional states across baseline and tasks

**Figure 3:**
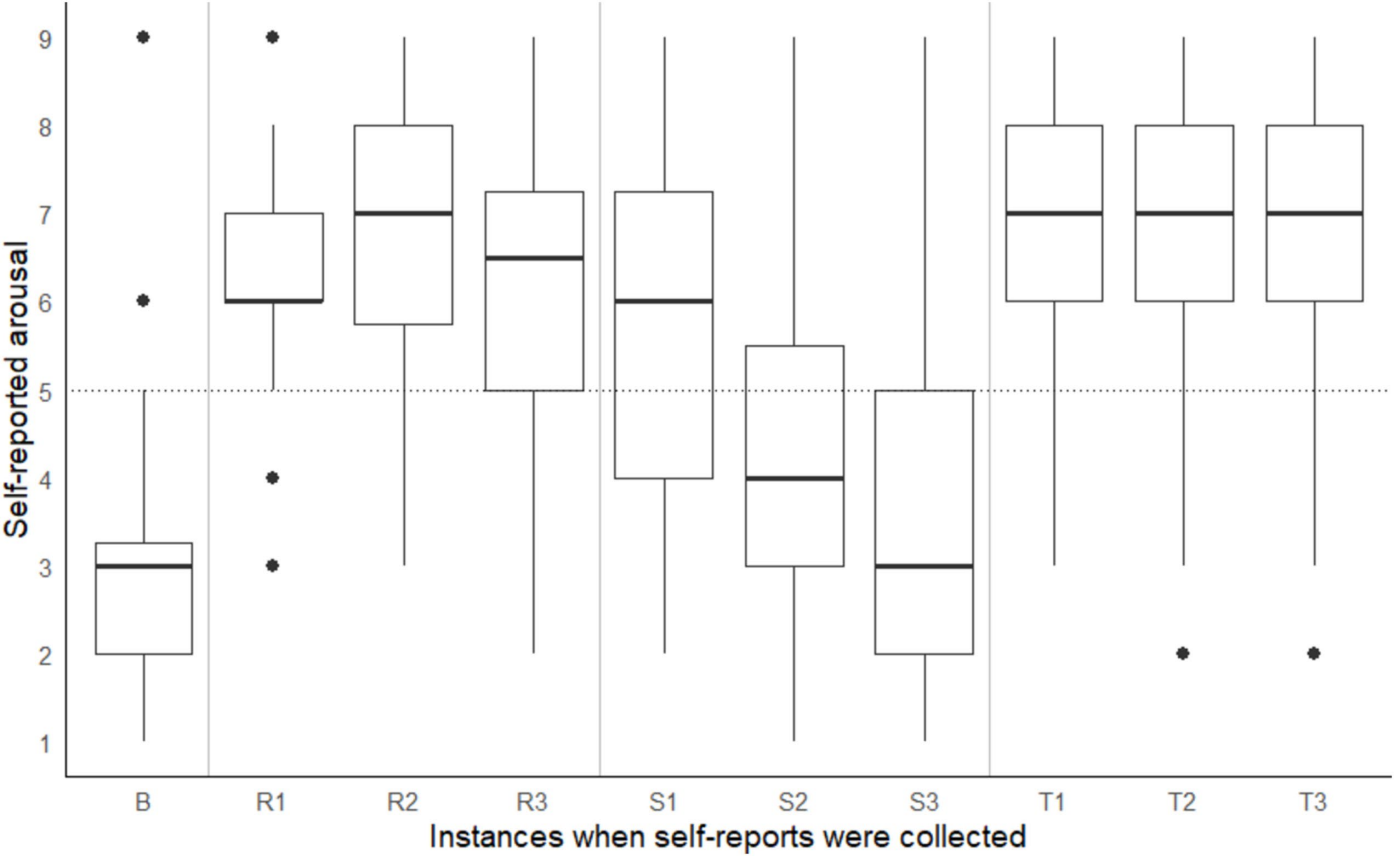
Self-reported arousal taken at baseline (B) and three points during the coordinate geometry puzzle (R1, R2, R3), shape-matching task (S1, S2, S3), and Tetris (T1, T2, T3). Note that task order was counterbalanced across participants.

**Figure 4:**
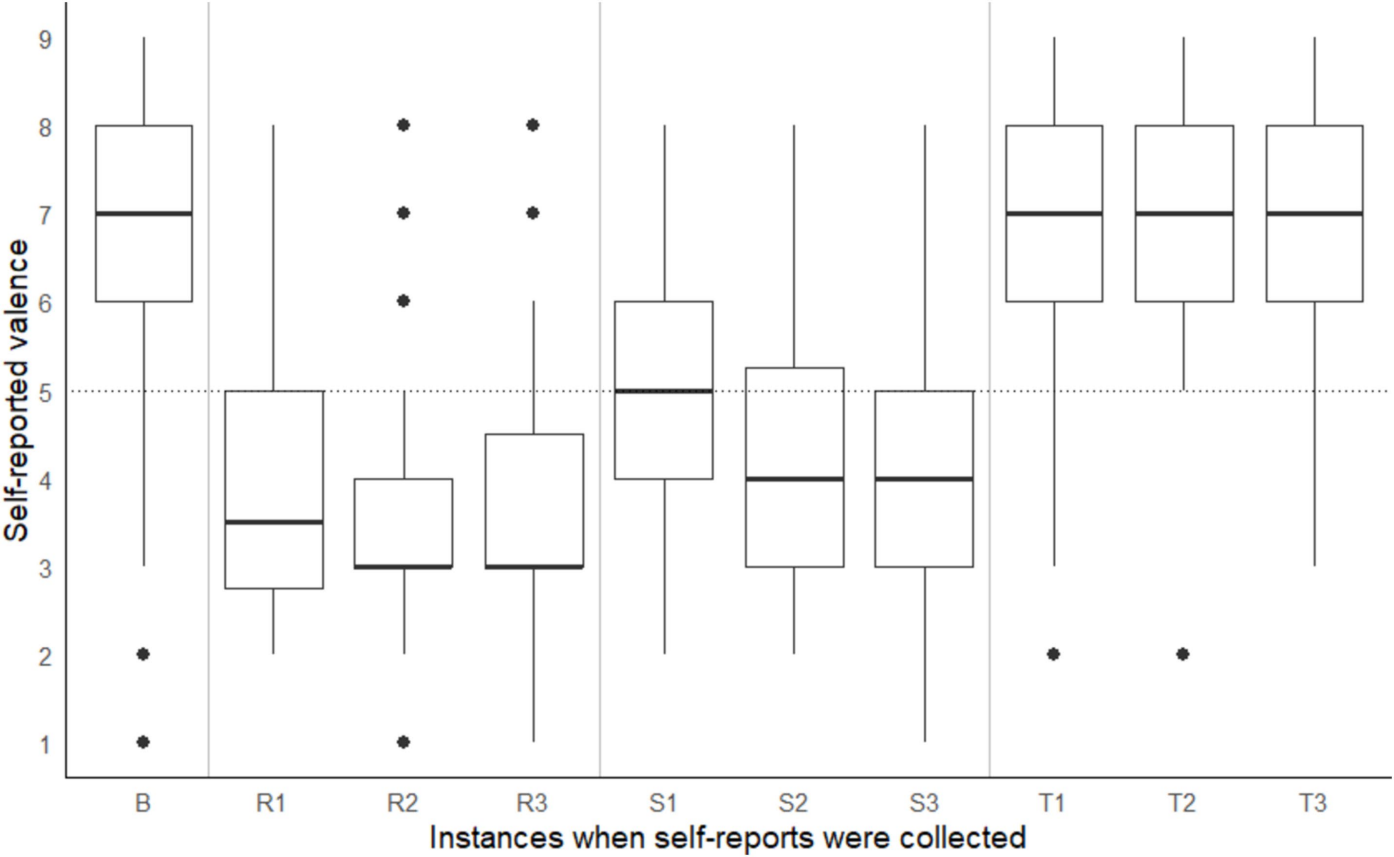
Self-reported valence taken at baseline (B) and three points during the coordinate geometry puzzle (R1, R2, R3), shape-matching task (S1, S2, S3) and Tetris (T1, T2, T3). Note that task order was counterbalanced across participants.

A significant within-subject difference in arousal and valence ratings across different points in time were observed, Pillai’s Trace = 0.94, *F*(18, 720) = 35.50, *p* < 0.001. On average, participants recorded the following Affect Grid values: (a) during the baseline reading, low arousal (rating < 5) and positive valence (rating > 5), (b) during the shape-matching task, a steady decline in arousal and pleasure (i.e., valence), (c) on the coordinate geometry puzzle, high arousal (rating > 5) and negative valence (rating < 5), and (d) on Tetris, high arousal (rating > 5) positive valence (rating > 5) (see Figures 3, 4). According to the open-ended fill-in-the-blank statements, most (34.1%) participants felt “relaxed” after the baseline. Most (22.2%) high arousal-negative valence ratings were accompanied by the word “frustrated” or “confused” (19.8%), followed by “nervous” (6.2%) or “annoyed” (6.2%). The most commonly used words used to supplement high arousal-positive valence ratings were “excited” (14.2%) or “happy” (12.8%), followed by “good” (10.6%) or “focused” (9.9%). Low arousal-positive valence ratings were accompanied by words such as “relaxed” (32.2%), “bored” (15.3%), “calm” (8.5%) and “sleepy” (6.8%). Lastly, most participants reported low arousal-negative valence when they felt “bored” (27.6%), “annoyed” (10.5%), “tired” (7.9%) or “sleepy” (7.9%).

### 3.2 Physiological and gaze features across emotional quadrants and pairwise differences

Kruskal-Wallis tests indicated no significant differences in SCR peak amplitude [χ^2^ = 6.57, df = 3, *p* = 0.09], average heart rate [χ^2^ = 5.58, df = 3, *p* = 0.13] and skin temperature [χ^2^ = 6.55, df = 3, *p* = 0.09]. However, tonic skin conductance levels [χ^2^ = 10.31, df = 3, *p* = 0.02], SCR peak count [χ^2^ = 24.99, df = 3, *p* < 0.001], fixation count [χ^2^ = 19.33, df = 3, *p* < 0.001], fixation duration [χ^2^ = 24.04, df = 3, *p* < 0.001], fixation dispersion [χ^2^ = 35.45, df = 3, *p* < 0.001], saccade count [χ^2^ = 29.33, df = 3, *p* < 0.001], saccade duration [χ^2^ = 33.36, df = 3, *p* < 0.001], saccade amplitude [χ^2^ = 26.92, df = 3, *p* < 0.001], blink count [χ^2^ = 31.25, df = 3, *p* < 0.001] and blink duration [χ^2^ = 42.34, df = 3, *p* < 0.001] showed variations across quadrants. Table 1 shows the significant pairwise differences obtained from *post hoc* comparisons using Dunn’s tests.

**Table 1:** Significant differences in features between quadrants of affect grid.

	Pairwise comparison of affect grid quadrants				
Feature	High arousal – Negative valence vs. High arousal – Positive valence	High arousal – Negative valence vs. Low arousal – Positive valence	High arousal – Negative valence vs. Low arousal – Negative valence	High arousal – Positive valence vs. Low arousal – Positive valence	High arousal – Positive valence vs. Low arousal – Negative valence
Tonic skin conductance			*p* = 0.005		
SCR peak count			*p* = 0.002		*p* < 0.001
Fixation count		*p* = 0.004	*p* = 0.006	*p* = 0.006	*p* = 0.009
Fixation duration		*p* = 0.009	*p* = 0.007	*p* = 0.002	*p* < 0.001
Fixation dispersion	*p* < 0.001				*p* < 0.001
Saccade count			*p* = 0.01	*p* = 0.002	*p* < 0.001
Saccade duration			*p* < 0.001	*p* = 0.01	*p* < 0.001
Saccade amplitude			*p* < 0.001	*p* = 0.02	*p* < 0.001
Blink count	*p* < 0.001	*p* = 0.01	*p* = 0.002		
Blink duration	*p* < 0.001				*p* < 0.001

Green: Group 1 > Group 2. For example, fixation duration in High Arousal – Negative Valence quadrant > Low Arousal – Positive Valence. Yellow: Group 1 < Group 2. For example, fixation count in High Arousal – Negative Valence quadrant < Low Arousal – Positive Valence.

## 4 Discussion

Emotions are integral to the learning experience. In this study, we sought to determine physiological and gaze indices of learner emotions with the goal of informing the design of learning systems that can adapt to the emotions of learners. Our findings indicate that when students experience emotions such as relaxation, characterized by low arousal and positive valence, they exhibit more and shorter fixations along with fewer blinks than when they experience high arousal-negative valence emotions such as frustration. Furthermore, low arousal-positive valence emotions differ from high arousal-positive emotions such as enjoyment in that they are accompanied by more and shorter fixations, fewer blinks, and more, slower and larger saccades. On the other hand, low arousal-negative emotions such as boredom differ from both high arousal-negative emotions and high arousal-positive emotions in that they are associated with fewer SCR peaks, more and shorter fixations, and more, slower and longer saccades. They are also characterized by longer blink durations and higher fixation dispersion than high arousal-positive emotions and fewer blink counts and lower tonic skin conductance than high arousal-negative emotions. Findings also indicate that high arousal-negative emotions are associated with higher fixation dispersion, blink count and blink duration as compared to high arousal-positive emotions. In fact, high arousal-negative emotions appear to be associated with the highest number of blinks. Heart rate and skin temperature were not found to be significant indicators of emotions.

High skin conductance observed during both positive and negative high-arousal emotions reaffirms the general understanding that this measure is a reliable index of emotional arousal (Boucsein, 2012). Meanwhile, examining the cognitive processes linked to each gaze measure and emotional state may provide possible explanations for the different eye movement patterns observed in this study. The high fixation counts observed during low-arousal emotions (e.g., boredom and relaxation) in this study reflect the findings of Foulsham et al. (2013) and Steindorf and Rummel (2019), that mindless reading can be associated with a high number of fixations. Earlier research has suggested that fixation count increases when “distractors are similar to targets” (Rayner, 1998), which may explain our findings, as boredom could lead to increased distraction, or in other words, increased attention to non-task-related elements. On the other hand, the high fixation durations observed during high-arousal emotions (such as frustration or excitement) in this study are likely due to increased visual attention and cognitive engagement, as these states often arise when individuals are task-oriented (Pekrun, 2006). Similarly, research on situational awareness—particularly in driving, where gaze dispersion is associated with heightened awareness of one’s surroundings—suggests that more dispersed gaze indicates greater situational awareness (Liang et al., 2021). This correlation may account for the high fixation dispersion observed during high-arousal, negative-emotional states when students in this study may have engaged in more extensive visual searches to solve a problem. In contrast, past results from a SART (Sustained Attention to Response Task) indicate that an attentive state is accompanied by higher fixation dispersion as compared to a mind wandering state (i.e., when one’s mind unconsciously wanders away from the task at hand) (Lee et al., 2021). Since mind wandering is a likely response to boredom (Randall et al., 2019), a low arousal-negative emotion, it may be possible to attribute this to the high fixation dispersion associated with this quadrant.

Additionally, several studies have found that eye movements during mind wandering are slower and less active than during attentive states (Faber et al., 2017; Uzzaman and Joordens, 2011). This is suggestive of slower and longer saccades associated with distracted visual scanning as compared to a focused visual pattern during task engagement. This is a possible explanation for the long saccade durations and large saccade amplitudes observed during low-arousal emotions (such as boredom or relaxation) in this study. Research on eye movements during stressful or anxiety-inducing situations, such as self-description in a foreign language, recalling a stressful event, viewing stress-or fear-inducing videos, and performing mental workload tasks (Giannakakis et al., 2017; Korda et al., 2021; Maffei and Angrilli, 2019) have shown a significant positive relationship between stress/anxiety and blink rate. The high blink rates observed during high-arousal, negative-valence emotions in this study are consistent with these findings. With respect to blink duration, research in vigilance and human factors suggests that longer blink durations during low-arousal, negative-valence states may be indicative of fatigue or drowsiness (Schleicher et al., 2008; Stern et al., 1996). This could explain this study’s findings of high blink duration during low arousal-negative emotions. Unfortunately, we do not have a possible explanation for the high saccade count during low arousal-negative emotions and large blink durations during high arousal-negative emotions.

In their review of eye-tracking metrics related to emotional and cognitive processes, Skaramagkas et al. (2023) highlighted the complex, non-linear relationship between gaze measures and emotions, a finding that aligns with the results of this study. Insights from this study draw attention to the importance of integrating multimodal data for emotion detection. Overall, our findings highlight the potential of physiological and gaze measures to distinguish between different learner emotions, thus paving the way for potential intervention moments when a learner moves from one emotional state to another.

Results notwithstanding, it is important to note the limitations of this study. Firstly, in this study, we were unable to differentiate between low arousal-negative emotions and low arousal-positive emotions. A possible explanation is the clustering of self-reported valence near-neutral in the third and fourth quadrants. Of the 98 ratings in these quadrants, 45 had valence values between 4 and 6, making it hard to distinguish between the groups of emotions. This is further reflected in the overlap of emotion labels, as the words “bored” and “sleepy” are associated with both quadrants. Secondly, despite all three tasks requiring some extent of visual scanning of a scene and visual selection of an object of interest, it is possible that (other) variability in the task demands influenced eye movement. This is potentially a confounding variable in our study and future studies can benefit from attempting to mitigate this by ensuring comparability of the visual demands of their tasks. It is also worth emphasizing that this study was conducted in a controlled environment to ensure the relevance of the signals, focusing on their applicability, rather than the sensors themselves. Though the current set-up seems distant from real-word classrooms, the rationale was to confirm the feasibility of these measures before applying them in a phased manner using more accessible sensors in classrooms. However, the controlled lab-setting of this study eliminated several distractions that one would normally find in a real classroom. Therefore, to run a similar study in real world classrooms would also require measures of students’ attention to the task at hand (thus ensuring that the emotions detected are in fact related to the learning) and error correction for external distractions. Additionally, words such as ‘annoyed’, ‘bored’ and ‘sleepy’ accompanied arousal and valence ratings in different quadrants of the Affect Grid. For example, sometimes participants experienced annoyance as a high arousal-negative emotion and sometimes as a low-arousal negative emotion. This is at odds with Russell’s (1980) circumplex model that places each emotion in one specific quadrant (in this case, annoyance as high arousal-positive valence emotion). It may be that persistence of annoyance of the former kind leads to other high arousal-negative emotions such as frustration or anger while the second kind leads to other low arousal-negative emotions such as hopelessness or gloominess. However, to provide a clear explanation for this is beyond the scope of this study. Lastly, we acknowledge that generalizability of this study’s findings are limited by the demographics included. For example, the sample primarily consisted of undergraduate students from Western Europe, which may not fully represent the broader population or account for physiological variations across different ages or cultural influences on emotional responses.

Mixed results of past research on what are reliable indicators of learner emotions may be attributed to a heterogeneity of (and sometimes a lack of transparency in) methodologies and devices used (Horvers et al., 2021; Lim et al., 2020; Yadegaridehkordi et al., 2019). With this paper, we hope to add to the corpus of clear methods for sensor-based studies in the field of education, thus paving the way for definitive study-design guidelines using such technology.

## 5 Conclusion

Students experience different emotions when engaging with learning-related tasks and this influences learning outcomes. Sensor technology today allows for (unobtrusive) collection of data that may eventually be used to provide personalized instruction or feedback to improve learning. In this paper, we investigated multimodal sensor data, namely skin conductance, skin temperature and gaze data as indicators of learner emotions operationalized by self-reports. Results indicate that skin conductance response peak count, tonic skin conductance levels, fixation count, duration and dispersion, saccade count, duration and amplitude, and blink count and duration can in fact be indicators of (self-reported) emotional arousal and valence in laboratory conditions. Researchers and designers may use these measures to make digital learning environments emotion-aware. These findings underline the need to move beyond the most extensively used measures – skin conductance and heart rate – and include several relevant factors. Results also reinforce the importance of doing psychophysiological research specific to the context of learning. On the whole, this study is a step toward emotion-aware learning systems.

## Data Availability

The processed data supporting the conclusions of this article will be made available by the authors, without undue reservation.
